# Proteolysis of MDA5 and IPS-1 is not required for inhibition of the type I IFN response by poliovirus

**DOI:** 10.1186/s12985-015-0393-2

**Published:** 2015-10-06

**Authors:** Swathi Kotla, Kurt E. Gustin

**Affiliations:** Present address: Department of Microbiology, Perelman School of Medicine, University of Pennsylvania, Philadelphia, PA 19104 USA; Department of Basic Medical Sciences, University of Arizona College of Medicine-Phoenix, Phoenix, AZ 85004 USA

**Keywords:** Poliovirus, Enterovirus, Interferon, IRF-3, MDA-5, Type I interferon, RIG-like receptors

## Abstract

**Background:**

The type I interferon (IFN) response is a critical component of the innate immune response to infection by RNA viruses and is initiated via recognition of viral nucleic acids by RIG-like receptors (RLR). Engagement of these receptors in the cytoplasm initiates a signal transduction pathway leading to activation of the transcription factors NF-κB, ATF-2 and IRF-3 that coordinately upregulate transcription of type I IFN genes, such as that encoding IFN-β. In this study the impact of poliovirus infection on the type I interferon response has been examined.

**Methods:**

The type I IFN response was assessed by measuring IFN-β mRNA levels using qRT-PCR and normalizing to levels of β-actin mRNA. The status of host factors involved in activation of the type I IFN response was examined by immunoblot, immunofluorescence microcopy and qRT-PCR.

**Results:**

The results show that poliovirus infection results in induction of very low levels of IFN-β mRNA despite clear activation of NF-κB and ATF-2. In contrast, analysis of IRF-3 revealed no transcriptional induction of an IRF-3-responsive promoter or homodimerization of IRF-3 indicating it is not activated in poliovirus-infected cells. Exposure of poliovirus-infected cells to poly(I:C) results in lower levels of IFN-β mRNA synthesis and IRF-3 activation compared to mock-infected cells. Analysis of MDA-5 and IPS-1 revealed that these components of the RLR pathway were largely intact at times when the type I IFN response was suppressed.

**Conclusions:**

Collectively, these results demonstrate that poliovirus infection actively suppresses the host type I interferon response by blocking activation of IRF-3 and suggests that this is not mediated by cleavage of MDA-5 or IPS-1.

## Introduction

Picornaviruses are small, positive sense, single-stranded RNA viruses belonging to the family *Picornaviridae*. The *Picornaviridae* now consists of nearly 30 different genera representing a diverse group of virus pathogens that cause disease in humans and animals. One of the most studied genera in this family is that of the *Enterovirus* which includes poliovirus, rhinovirus and coxsackievirus among others. Following release of viral RNA into the cytoplasm the viral genome is translated into a single polyprotein, which is proteolyzed to produce individual viral proteins responsible for RNA synthesis, assembly and modulation of host cell functions. RNA synthesis is carried out by the virus-encoded RNA-dependent RNA polymerase that first transcribes the plus-strand to produce a double stranded RNA (dsRNA) consisting of full length plus and minus-strand RNAs and known as the replicative form (RF-RNA). Newly synthesized minus-strands serve as a template for plus-strand synthesis and result in the appearance of full length plus-strands along with replicative intermediates consisting of incomplete plus strands partially annealed with the minus strand (Reviewed in [1]).

Recognition of viral RNA species in infected cells results in the transcriptional activation of the Type I interferon (IFN) response. Viral dsRNA is recognized by membrane bound and cytosolic cellular pattern recognition receptors. Cytosolic receptors include the RIG-like-Receptors, RIG-I and MDA-5 that signal through the adapter protein IPS-1 (also known as Cardiff, VISA or MAVS) (Reviewed in [2]). Membrane bound receptors include the Toll-like receptor, TLR3, which recognizes dsRNA in the endosomal compartment (Reviewed in [3]). Activation of RLRs and TLRs initiates distinct signaling pathways that converge on the cellular transcription factors NF-κB, IRF-3 and ATF-2 which are required for the induction of IFN-β mRNA and the type I interferon response [4]. Secreted IFN-β binds to the type I IFN receptor to activate the Jak/STAT signaling pathway [5] resulting in the production of a variety of proteins having antiviral, immunomodulatory and antiproliferative functions [6].

The RIG-like-receptor (RLR) Melanoma differentiation-associated gene 5 (MDA-5) is thought to be critical for the recognition of picornavirus RNA based on the observation that mice lacking MDA-5 are more susceptible to encephalomyocarditis virus, another picornavirus [7, 8]. Subsequent work using siRNA knockdown or mouse embryonic fibroblasts lacking MDA-5 has shown that recognition of the double stranded RF-RNA is critical for induction of type I IFN in tissue culture [9–11]. More recent work found that TLR3 plays an important role in modulating the host response to poliovirus-infection in a transgenic mouse model [12, 13]. Thus, it appears that multiple pathways may contribute to limiting pathogenesis associated with enterovirus infections.

Work done in the late 1950s showed that poliovirus replication is sensitive to the antiviral effects of type I interferon in tissue culture [14]. More recent work in transgenic mice expressing the poliovirus receptor, CD155, has extended these finding by showing that the type I interferon response plays a critical role in controlling disease progression by inhibiting replication in non-neural tissue and preventing spread to the central nervous system [15, 16]. While these findings indicate that IFN may be effective against poliovirus, more recent work has shown that poliovirus can overcome the inhibitory effects of this antiviral cytokine. For example, poliovirus can replicate in tissue culture cells exposed to low doses of interferon and this is dependent upon activities provided by the virus 2A protease [17].

In addition to overcoming the antiviral effects of IFN, several findings suggest that poliovirus inhibits pathways leading to the initial synthesis of IFN-β. Although enterovirus RF-RNA is a potent activator of IFN-β synthesis when transfected into cells [9, 10] very little IFN-β is actually made in poliovirus, or other enterovirus-infected cells suggesting that activation of this pathway may be impaired [18–22]. Also in support of this possibility is the finding that little or no activated IRF-3 is detected in cells infected with rhinovirus, enterovirus71 or coxsackievirus B3 [19–23]. Furthermore, both human rhinovirus 1a and coxsackievirus B3 block the ability of sendai virus to activate IRF-3 indicating that these enteroviruses can actively suppress induction of the type I IFN response pathway [20, 21]. An explanation for these findings was provided by the observation that RIG-I, MDA-5 and IPS-1 are proteolyzed during infection with these viruses [21–25]. MDA-5, RIG-I and IPS-1 are also proteolyzed in poliovirus-infected cells [21, 24, 25] although the status of the type I IFN response pathway has not been examined.

To address this, we have examined the induction of IFN-β mRNA and the activation status of NF-κB, IRF-3 and ATF-2 in poliovirus-infected cells. The results demonstrate that poliovirus infection results in the activation of both ATF-2 and NF-κB but very little activation of IRF-3. In addition, the ability of infected cells to synthesize IFN-β mRNA in response to stimulation with poly(I:C) is impaired indicating that the type I IFN response is suppressed. Surprisingly, inhibition occurs before significant degradation of NF-κB, MDA-5 or IPS-1 is apparent in infected cells. These results suggest that poliovirus, and perhaps other enteroviruses, may target additional host factors to mediate inhibition of the host type I interferon response.

## Results

### Poliovirus infection fails to activate the type I IFN response

**Fig. 1:**
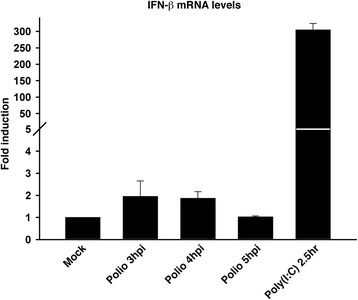
Poliovirus infection fails to induce a type I interferon response in HEC-1B cells. Total RNA extracted from uninfected, poliovirus-infected or poly(I:C) treated cells at the indicated times post-infection was analyzed by qRT-PCR with primers for both IFN-β and β-actin mRNA. The amount of IFN-β mRNA was normalized to the amount of β-actin mRNA. The fold induction of IFN-β mRNA relative to uninfected controls is shown. Error bars indicate the standard error of the results from triplicate wells. hpi: hours post infection

Το examine the status of the type I IFN response following infection with poliovirus we selected HEC-1B cells. HEC-1B cells are non-responsive to interferon due to the lack of functional IFNα/ IFNβ receptors [26, 27]. Consequently the induction of type I interferon in this cell line is entirely due to the infecting virus and independent of autocrine or paracrine signaling that would further amplify the cellular response. Prior work using RNase protection assays had reported little accumulation of IFN-β mRNA in HEC-1B cells following infection [18]. To quantitatively assess IFN-β mRNA levels in poliovirus-infected HEC-1B cells RNA was isolated and analyzed by qRT-PCR. Poliovirus-infected cells showed only a 2 fold increase in IFN-β mRNA levels at 3 and 4 h post infection (hpi) and by 5 hpi levels were very similar to mock-infected controls (Fig. 1). As a positive control we treated the cells with poly(I:C), a synthetic analogue of double-stranded RNA (dsRNA) and a known inducer of IFN-β mRNA and the type I IFN response [28]. Figure 1 shows that poly(I:C) treatment of HEC-1B cells resulted in greater than 300 fold induction of IFN-β mRNA compared to the mock-infected controls. In agreement with other reports, low levels of IFN-β mRNA were also observed in poliovirus-infected HeLa cells, although levels were higher compared to what was observed in poliovirus-infected HEC-1B cells, perhaps due to amplification via the Jak/STAT pathway (data not shown) [24, 29]. Poliovirus RNA levels increased by 4 logs from 1 to 5 hpi confirming active replication in this cell line as reported by Dodd et al. ([18] and data not shown). These results demonstrate that poliovirus infection does not induce a strong type I interferon response in either HeLa or HEC-1B cells.

### Poliovirus infection inhibits the ability of cells to respond to dsRNA

**Fig. 2:**
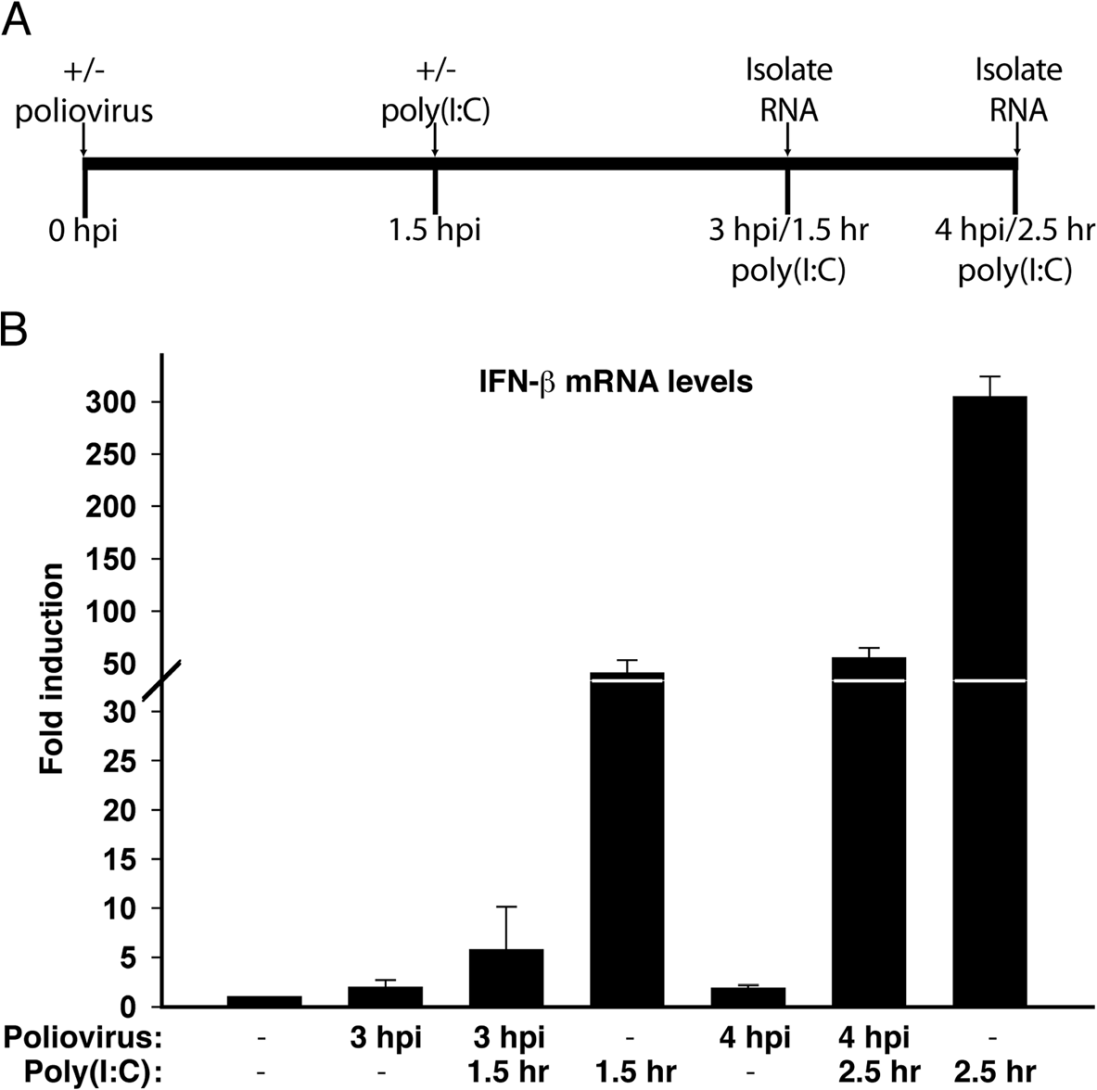
Poliovirus infection inhibits the ability of cells to respond to dsRNA. **a**. Schematic outlining the experimental design. At zero hours cells were either mock infected or infected with poliovirus at an MOI of 100 and 1.5 h later either treated or not with poly(I:C). After another 1.5 h (3 hpi) or 2.5 h (4 hpi) RNA was extracted for analysis by qRT-PCR. **b**. IFN-β mRNA levels in poliovirus-infected HEC-1B cells treated as described above. Total RNA was isolated and analyzed by qRT-PCR for IFN-β mRNA and normalized to levels of β-actin mRNA. The fold induction of IFN-β mRNA relative to uninfected controls is shown. Error bars indicate the standard error of the results from triplicate wells. hpi: hours post infection

The lack of induction of IFN-β mRNA in poliovirus-infected cells indicates that the virus might interfere with the activation of this host defense signaling pathway. Consistent with this possibility, Dodd and Kirkegaard used RNase protection assays to show that poliovirus inhibits the ability of cells to produce IFN-β mRNA in response to treatment with poly(I:C) [30]. To further validate and quantify the extent of inhibition, we have analyzed IFN-β mRNA levels in poliovirus-infected cells treated with poly(I:C) by qRT-PCR. Previous studies have shown that poliovirus infection begins to inhibit host cell transcription at ~3 hpi with maximal inhibition not occurring until 5 hpi [31, 32]. Hence, we have conducted these experiments at early times following infection to reduce complications from poliovirus-mediated host transcriptional shut off. The strategy used for this analysis is outlined in Fig. 2a. Briefly, HEC-1B cells were infected with poliovirus and then treated with poly(I:C) at 1.5 h post-infection. RNA was isolated after an additional 1.5 or 2.5 h (3 and 4 hpi, respectively) and the levels of IFN-β mRNA were determined. When uninfected cells were treated with poly(I:C) IFN-β mRNA was induced 40-fold after 1.5 h and 305-fold after 2.5 h (Fig. 2b). However, if cells were first infected with poliovirus and then treated with poly(I:C) the level of induction after 1.5 h (3 hpi) was only 6-fold and after 2.5 h (4 hpi) only 55-fold. Quantification of these results revealed that poliovirus inhibited the amount of IFN-β mRNA produced in response to poly(I:C) treatment for 1.5 and 2.5 h by 85 % and 82 %, respectively. Since poliovirus inhibits host cell transcription by only 35 % at 3 hpi this inhibition is likely not entirely due to the host transcriptional shut off in infected cells. Although poliovirus infection results in the inhibition of host protein synthesis, previous studies have indicated that *de novo* protein synthesis is not required for the induction of IFN-β mRNA [33]. This indicates that the poliovirus-mediated inhibition of IFN-β induction is not likely due to host translational shutoff and suggests that poliovirus infection specifically inhibits the ability of cells to sense and/or respond to dsRNA.

### Status of ATF-2 and NF-κB in poliovirus-infected cells

Transcriptional induction of the IFN-β promoter requires the coordinated action of the activated transcription factors ATF-2, NF-κB and IRF-3. To determine the reason for the lack of induction of IFN-β mRNA in poliovirus-infected cells the status of these transcription factors was analyzed.

**Fig. 3:**
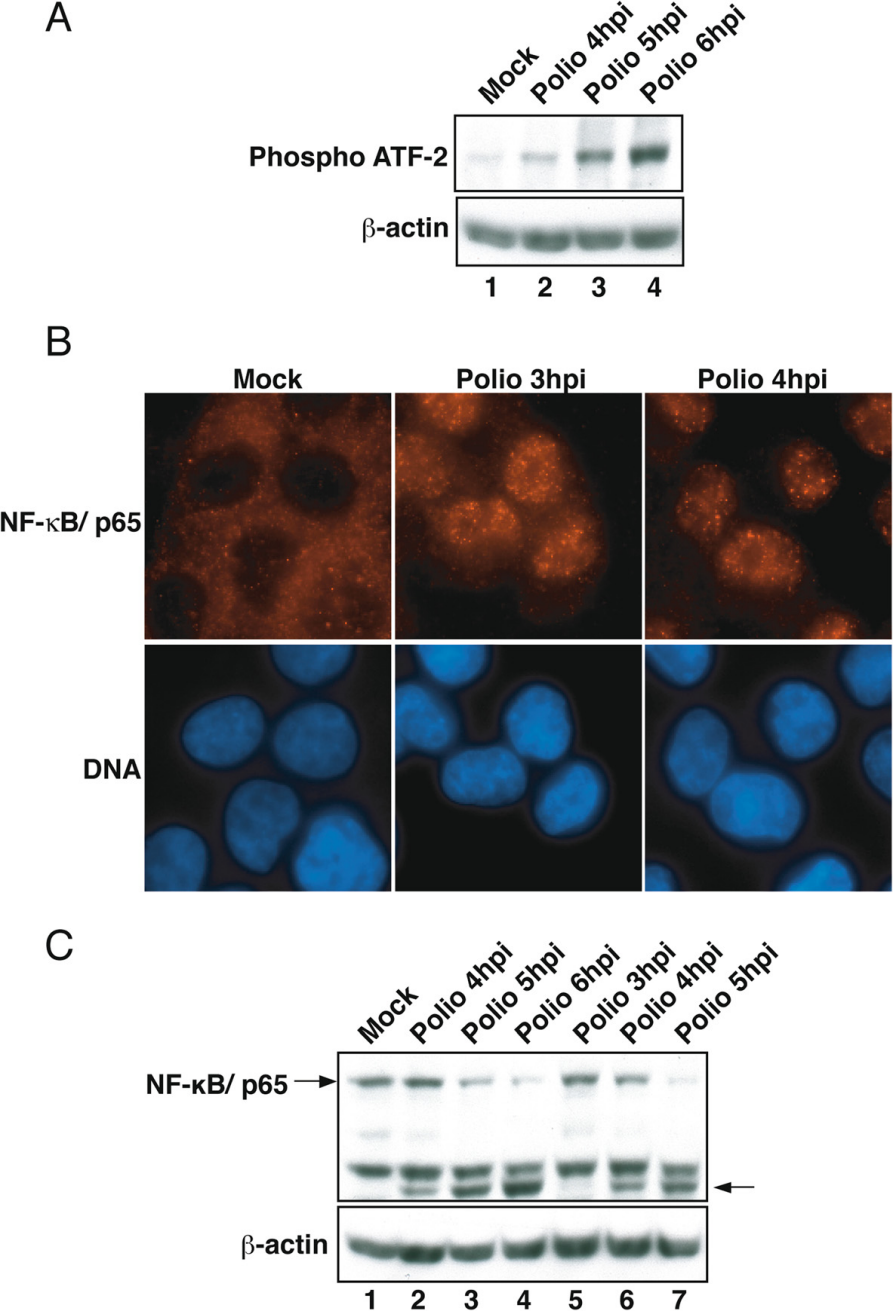
ATF-2 and NF-κB are activated in poliovirus-infected cells. **a**. Phosphorylation of ATF-2. Whole cell lysates prepared from uninfected or poliovirus-infected cells were analyzed by immunoblotting. Phosphorylated ATF-2 (phospho-ATF-2) was detected by using an antibody specific for the phosphorylated form of ATF-2. The membrane was also probed with an antibody to β-actin to show equivalent loading of protein lysates. **b**. Subcellular localization of NF-κB. Cells that were mock-infected or had been infected with poliovirus for the indicated amount of time were analyzed by immunofluorescence with antibodies to detect the p65 subunit of NF-κB. Panels labeled NF-κB show cells stained with a rabbit polyclonal antibody to detect the p65 subunit of NF-κB using TRITC filter, while the panels labeled DNA show the same fields stained with Hoechst to reveal the nuclei. **c**. Steady state levels of NF-κB. Whole cell lysates prepared from uninfected or poliovirus-infected cells at an MOI 50 (lanes 2–4) or 100 (lanes 5–7) were analyzed by immunoblotting. The p65 subunit of NF-κB was detected by probing the membrane using antibody specific for p65. The membrane was also probed with an antibody to β-actin to show equivalent loading of protein lysates. Arrows indicate cleavage products produced following infection. hpi: hours post infection

ATF-2 resides in the nucleus at steady state and following activation gets phosphorylated on two threonine residues [34]. To determine if ATF-2 is activated in poliovirus-infected cells a phospho-specific antibody that recognizes activated ATF-2 was used. As expected, very little phospho-ATF-2 was present in mock-infected cells consistent with ATF-2 being in an inactivated state (Fig. 3a, lane 1). Following infection the amount of phospho-ATF-2 was elevated at 4 hpi and continued to increase through 6 hpi (Fig. 3a, lanes 2–4). These results indicate that ATF-2 is intact and activated in poliovirus-infected cells.

NF-κB normally resides in the cytoplasm, but following infection by many viruses NF-κB is activated and rapidly accumulates in the nucleus [35, 36]. Neznanov et al., found that NF-κB is activated following infection by poliovirus and then subsequently degraded [37]. Thus it was possible that proteolysis of NF-κB might be responsible for the lack of IFN- β transcription in poliovirus-infected cells. Consequently, we examined the sub-cellular localization and the steady state levels of NF-κB in poliovirus-infected HEC-1B cells. Figure 3b shows that, as expected, NF-κB is cytoplasmic in mock-infected cells and that by 3 hpi and 4 hpi it is predominantly nuclear, suggesting that NF-κB is activated in HEC-1B cells following infection with poliovirus. To determine if NF-κB is cleaved in poliovirus-infected HEC-1B cells, steady state levels of the protein were examined. Immunoblot analysis shows that although proteolysis is apparent at 4 hpi there is still a significant amount of intact NF-κB remaining in infected cells at this time (Fig. 3c, lanes 2). Even at an MOI of 100 NF-κB levels were unchanged at 3 hpi although levels had declined significantly by 4 and 5 hpi (Fig. 3c, lanes 5–7). These results are in agreement with the previously published analysis of NF-κB in poliovirus-infected HeLa cells [37]. The finding that NF-κB is predominantly nuclear and is mostly intact at 3 hpi suggests that inhibition of the type I IFN response is not due to proteolysis of this transcription factor.

### Poliovirus infection fails to activate IRF-3

**Fig. 4:**
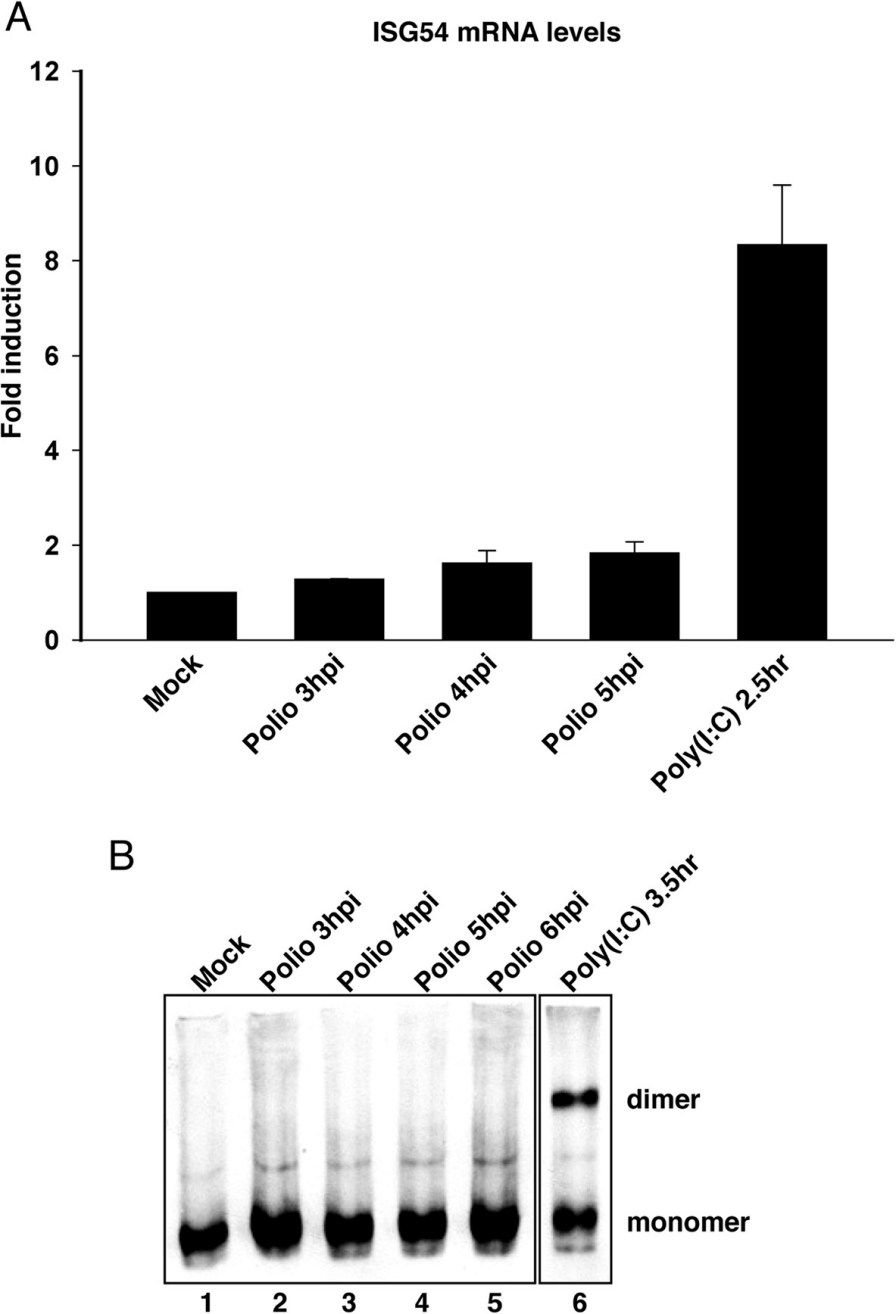
IRF-3 is not activated in poliovirus-infected cells. **a**. ISG54 mRNA levels in infected cells. Total RNA extracted from uninfected, poliovirus-infected or poly(I:C) treated cells at the indicated times post-infection was analyzed by qRT-PCR with primers for both interferon stimulated gene 54 (ISG54) and β-actin mRNAs. The amount of ISG54 mRNA was normalized to the amount of β-actin mRNA. The fold induction of ISG54 mRNA relative to uninfected controls is shown. Error bars indicate the standard error of the results from triplicate wells. hpi: hours post infection. **b**. Homodimerization of IRF-3. Whole cell lysates prepared from cells that were mock-infected or had been infected with poliovirus for the indicated amount of time or treated with poly(I:C) for 3.5 h were analyzed by native PAGE followed by immunoblot to detect IRF-3. The monomeric and dimeric forms of IRF-3 are indicated

To determine if IRF-3 is transcriptionally active in poliovirus-infected cells we have examined the induction of interferon stimulated gene 54 (ISG54), an IRF-3 responsive transcript [38]. Figure 4a shows that the levels of ISG54 mRNA in poliovirus-infected cells were very similar to the mock-infected controls even at 4 and 5 hpi. In contrast, poly(I:C), an inducer of IRF-3 and the type I IFN response resulted in a significant induction of ISG54. The low levels of IFN-β and ISG54 mRNAs, suggest that IRF-3 activation may be impaired in poliovirus-infected cells.

Transcriptional activation by IRF-3 requires phosphorylation of S/T residues located in the C-terminus leading to nuclear translocation and dimerization [39]. Consequently, the dimerization status of IRF-3 was determined in poliovirus-infected cells by native PAGE followed by immunoblot. Separation of lysates on non-denaturing PAGE allows differentiation between the dimeric activated and monomeric inactive forms of IRF-3 [40]. As expected, dimeric IRF-3 was not seen in mock-infected cells, but was readily detected following poly(I:C) treatment for 3.5 h (Fig. 4b, lanes 1 and 6). Analysis of lysates from poliovirus-infected cells failed to reveal detectable IRF-3 homodimer even at 5 and 6 hpi (Fig. 4b, lanes 4 and 5). The lack of induction of ISG54 and IFN-β mRNA along with undetectable IRF-3 dimer suggests that IRF-3 is not activated in poliovirus-infected cells.

### Poliovirus infection inhibits poly(I:C) induced IRF-3 activation

**Fig. 5:**
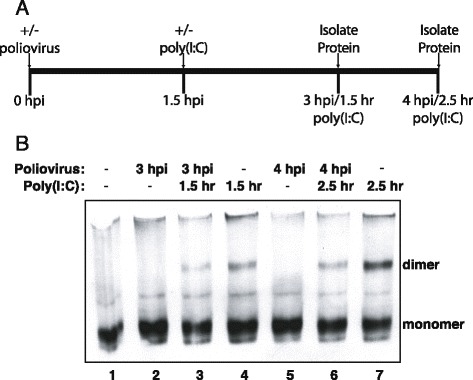
Poliovirus infection inhibits poly(I:C) induced IRF-3 activation. **a**. Experimental design. At zero hours cells were either mock infected or infected with poliovirus at an MOI of 100 and at 1.5 h following infection or mock infection cells were either treated or not with poly(I:C). After another 1.5 h/ 2.5 h lysates were harvested to extract protein. **b**. HEC-1B cells were either mock-infected or infected with poliovirus at an MOI of 100 and then treated with poly(I:C) or not for the indicated amount of time. Whole cell lysates were prepared in non-denaturing lysis buffer and were analyzed by native PAGE followed by immunoblot to detect IRF-3. The monomeric and dimeric forms of IRF-3 are indicated. hpi: hours post infection

Several picornaviruses, including enteroviruses have been shown to inhibit activation of IRF-3 in response to exogenous stimuli [20, 21, 41]. As the results of Fig. 2b indicated that poliovirus can inhibit the cellular response to poly(I:C), we wanted to determine if this might be attributed to a viral blockade of IRF-3 activation. To see if this was the case, IRF-3 dimerization was assayed in lysates from poliovirus-infected cells that had been treated with poly(I:C). As expected there was good induction of IRF-3 dimer in uninfected cells treated with poly(I:C) for 1.5 or 2.5 h (Fig. 5, lanes 4 and 7) and IRF-3 dimer was undetectable in cells infected with poliovirus (Fig. 5, lanes 2 and 5). Analysis of cells infected and then treated with poly(I:C) revealed that although IRF-3 homodimers were detected, their levels were reduced compared to cells treated with poly(I:C) alone (Fig. 5, compare lane 3 with 4 and lane 6 with 7). These results suggest that the inhibition of IFN-β mRNA production observed in poliovirus-infected cells treated with poly(I:C) may be mediated by disrupting IRF-3 activation.

### Status of MDA-5 and IPS-1 in poliovirus-infected cells

**Fig. 6:**
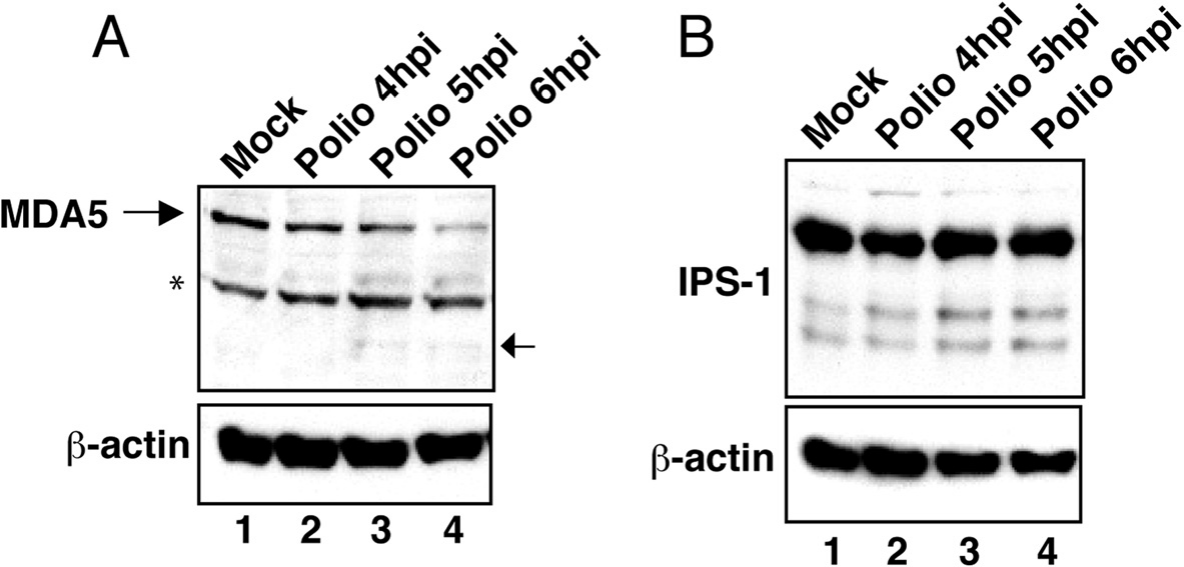
Steady state levels of MDA-5 and IPS-1. Whole cell lysates prepared from cells that were mock-infected or had been infected with poliovirus for the indicated amount of time were analyzed by immunoblotting with rabbit polyclonal antibodies that detect MDA-5 (**a**) or IPS-1 (**b**). The membrane was stripped and reprobed with an antibody to β-actin to show equivalent loading of protein lysates. (*) indicates nonspecific band; Arrow indicates cleavage product

Prior studies have suggested that enteroviruses can disrupt the activation of IRF-3 by causing the degradation of key signaling molecules such as MDA-5 and IPS-1 that are required for its activation [20–25]. Analysis of poliovirus-infected HeLa cells cells found that proteolysis of MDA-5 and IPS-1 was not apparent until 4 hpi or later [21, 24], making it possible that proteolysis of MDA-5 and IPS-1 occurs after the inhibition of the type I IFN response reported here. Consequently, we examined MDA-5 and IPS-1 in infected HEC-1B cells to determine if their proteolysis might explain the lack of IRF-3 activation. Analysis of steady state levels of MDA-5 protein in infected HEC-1B cells shows that even at 5 hpi very little cleavage of MDA-5 is detectable although it becomes more apparent at later times (Fig. 6a, lanes 2–4). The presence of significant amounts of intact MDA-5 protein at 4 hpi makes it seem unlikely that cleavage of this protein contributes to the observed inhibition of IRF-3 dimerization seen in infected cells at 3 and 4 hpi. A similar analysis of IPS-1 in poliovirus-infected HEC-1B cells reveals very little evidence of proteolysis even at 6 hpi (Fig. 6b, lane 4). These results indicate that the inhibition of IRF-3 activation in poliovirus-infected HEC-1B cells does not appear to be mediated by the proteolytic cleavage of MDA-5 or IPS-1.

## Discussion

This study has examined the induction of the type I interferon response and the activation status of IRF-3, NF-κB and ATF-2 in poliovirus-infected HEC-1B cells. The results confirm that only very low levels of IFN-β mRNA are induced following poliovirus infection and suggest that the ability of infected cells to mount a type I IFN response is impaired [18, 29]. Analysis of the transcription factors required for induction of IFN-β mRNA synthesis revealed that both NF-κB and ATF-2 were present and activated in infected cells indicating that HEC-1B cells can sense and respond to poliovirus-associated molecular patterns. Despite this, IRF-3 is not activated in poliovirus-infected cells as evidenced by the lack of detectable levels of IRF-3 homodimer and transcriptional induction from the *ISG54* promoter. The finding that poliovirus is able to inhibit poly(I:C) induced IRF-3 homodimerization and IFN-β mRNA synthesis indicates that poliovirus actively suppresses the host response to dsRNA. Notably, inhibition occurs at a time when IPS-1 and MDA-5 are not significantly degraded suggesting alternative mechanisms are responsible for the blockade in IRF-3 activation seen in poliovirus-infected cells.

Several picornaviruses are known to inhibit the type I IFN response. HRV1a, coxsackievirus B3 and hepatitis A virus have been shown to block the IRF-3 activation normally seen in cells infected with sendai virus [20, 21, 42] and mengovirus infection blocks IRF-3 dimerization in response to poly(I:C) [41]. In poliovirus-infected cells MDA-5, RIG-I and IPS-1 are all cleaved by viral and/or cellular proteases providing a potential mechanism for inhibition of the type I IFN response [21, 23–25]. However, analysis of infected cells reveals that cleavage of these proteins does not begin until 4 hpi or later [21, 24, 25]. The results presented here from HEC-1B cells are consistent with these earlier findings and show that both IPS-1 and MDA-5 are mostly intact at 4 hpi. Significantly, cells at this time are unable to mount a type I IFN response when transfected with poly(I:C). Cumulatively, the results suggest that poliovirus targets other factors in the type I IFN response pathway to inhibit the synthesis of IFN- β mRNA at early times post-infection.

Recognition of viral RNA by MDA-5 and signaling via IPS-1 clearly plays an important role in host defense to picornaviruses under certain conditions [9, 10, 13]. However, studies in transgenic animals expressing the poliovirus receptor (PVR) suggest that other pathways are crucial for protection from infection in vivo. These studies found that PVR-transgenic mice lacking MDA-5, RIG-I or IPS-1 are no more susceptible to poliovirus-induced disease than wild type mice [12, 13]. In contrast, mice lacking TLR3 or the TLR3-specific adaptor molecule, Toll/IL-1R homology domain-containing adaptor molecule 1 (TICAM-1, also known as TRIF) exhibited increased mortality compared to wild-type littermates when challenged with low infectious doses of poliovirus [12]. These results suggest that recognition of poliovirus by the TLR3 pathway is critical for protection from disease in vivo and suggest that it would be advantageous for poliovirus to target components of the TLR3 pathway to inhibit type I IFN synthesis. Evidence supporting this possibility comes from studies in coxsackievirus-infected cells showing that both RLR and TLR3 pathways are compromised. Mukherjee et al., found that in CVB3-infected HEK293 cells both IPS-1 and TICAM-1 are proteolyzed [22]. Of note, this study found that cleavage of TICAM-1 occurs earlier and is more extensive than that of IPS-1 in CVB3-infected cells perhaps providing a clue as to the relative or temporal importance of these pathways in controlling CVB3 infection. Similarly, enterovirus 68 infection inhibits the ability of HeLa cells to respond to poly(I:C) and this is associated with cleavage of TICAM-1 and reduced IRF-3 activation [43]. Clearly experiments examining the status of the TLR3 pathway in poliovirus-infected cells are warranted.

The finding that NF-κB is activated in infected cells indicates that the sensing and initial signaling response is intact at least until the NF-κB and IRF-3 activation pathways diverge. Following activation of MDA-5 by dsRNA it interacts with IPS-1 via common caspase activation and recruitment domains (CARD) and promotes IPS-1 aggregation and activation on the mitochondrial membrane [44]. Activated IPS-1 then serves as a platform for recruitment of TRAF-6 and ultimately activation of the NF-κB kinase complex IKKα/β and the IRF-3 kinases TBK1 and IKK-ε [44]. Similarly, The TLR3 pathway, following the recruitment of TICAM-1/TRIF via common Toll/IL-1 receptor (TIR) domains, leads to activation of these same kinases [3]. Thus the TBK1 and IKK-ε kinases responsible for the ultimate phosphorylation and activation of IRF-3 and common to both pathways would appear to be possible targets in poliovirus-infected cells. Although analysis of CVB3-infected cells indicate TBK1 is not activated or targeted for proteolysis during infection with this enterovirus [23] the status of TBK-1 or IKK-ε in poliovirus-infected cells has not been examined.

Infection with many viruses, including poliovirus [45], induces the formation of cytoplasmic structures called stress granules (SG) that are composed of RNAs and host RNA binding proteins and have been implicated in the anti-viral response [46]. Although the mechanism by which SGs inhibit viral replication is not entirely understood recent reports indicate SGs may have a role in modulating the type I IFN response. For example, the NS1 protein of influenza is a potent inhibitor of the type I IFN response and little IFN is produced, or SGs formed, in cells infected with wild type influenza [47]. However, in cells infected with a mutant influenza virus lacking the NS1 protein strong induction of IFN-β mRNA is seen along with SGs that contain RIG-I, MDA-5 and IPS-1 [47]. Similarly, cells infected with encephalomyocarditis virus (EMCV) or a mutant Theiler’s murine encephalomyelitis virus that activates a type I IFN response also caused SG formation along with recruitment of MDA-5 to these structures [48, 49]. Notably, SG formation was inhibited in cells lacking the dsRNA activated kinase, PKR and PKR was also found to be recruited to SGs in infected cells [47–49]. In the case of EMCV, influenza and coxsackievirus blocking SG formation during infection impairs the ability of cells to induce IFN-β mRNA synthesis [47, 49, 50]. These results directly link SG formation to activation of the type I IFN response by certain viruses, including picornaviruses, although one report indicates this may not always be the case [48].

Both poliovirus and EMCV inhibit the anti-viral activities of SGs by proteolyzing the cellular RAS GAP SH3-domain binding protein 1 (G3BP), a key component of SGs [49, 51]. Consistent with the antiviral nature of SGs, blocking the ability of poliovirus or EMCV to cleave G3BP and inhibit SGs reduces virus yields obtained from infected cells [49, 51]. Furthermore, blocking G3BP cleavage in EMCV-infected HeLa cells allows SG formation and results in elevated levels of IFN- β [49]. Thus it seems likely that modulation of SG nucleation and composition may contribute to the block in IRF-3 activation and inhibition of the type I IFN response seen in poliovirus-infected cells. While this may be the case, additional experiments will be needed to determine if G3BP cleavage and its effect on SG activity interferes with IRF-3 activation and if so, how this occurs without affecting activation of NF-κB and ATF-2.

Another intriguing possibility comes from work showing that EV71 modulates the levels of TRAF6 and IRAK1 during infection. TRAF6 is a ubiquitin ligase that has an important role in signaling following TLR and RLR engagement, while IRAK1 is involved primarily in signaling from TLR7 and 9 [3, 44]. EV71 up regulates expression of miR146a that, in turn, suppresses translation of IRAK1 and TRAF6 mRNAs [52]. Blocking the action of miR146a restores levels of IRAK1 and TRAF6 in infected cells, increases IFN- β mRNA levels and improves survival in a mouse model of EV71 pathogenesis [52]. Cells infected with poliovirus and coxsackievirus also show elevated levels of miR146a and reduced expression of IRAK1 and TRAF6 indicating they may employ a similar mechanism to impede type I IFN synthesis [52]. While targeting TRAF6 and IRAK1 would interfere with the Type I IFN response initiated by both RLR and TLR signaling it would also be expected to inhibit NF-kB activation. Thus, as is the case for SGs described above, how miRNA-mediated inhibition of IRAK1 and TRAF6 by poliovirus selectively interferes with IRF-3 activation without impeding NF-κB or ATF-2 activation remains to be determined.

## Materials and methods

### Cell culture and viruses

HEC-1B cells were grown in monolayer in Dulbecco’s Modified Eagle’s Medium (DMEM) supplemented with 10 % Fetal Bovine Serum (FBS), 2 mM L-Glutamine and Penicillin-Streptomycin at 37 °C in 5 % CO_2_. Mahoney type I poliovirus (PV) stocks were prepared by infecting sub-confluent HeLa monolayers at a multiplicity of infection (MOI) of 10. Virus was adsorbed for 30 min at 37 °C in CPBS (phosphate-buffered saline (PBS) supplemented with 1 mM MgCl_2_ and 1 mM CaCl_2_). Following adsorption, residual virus was removed and complete medium was added. At 7 h post infection (hpi), cells were scraped into CPBS, pelleted at 1500 × *g*, resuspended in CPBS and subjected to three freeze and thaw cycles followed by centrifugation at 10,000 × *g* for five minutes at 4 °C. The supernatant was titered on HeLa cells, aliquoted and stored at −70 °C. Unless otherwise indicated sub-confluent HEC-1B cells were infected with poliovirus at an MOI of 50 as described above.

### RNA isolation and cDNA synthesis

Mock and infected cells were incubated for the indicated amounts of time and total RNA was prepared using the Qiagen RNeasy mini kit (Qiagen; 74104). To remove any traces of contaminating DNA, on column DNA digestion was carried out using RNase-Free DNase kit (Qiagen; 79254) as described by the manufacturer. The quantity and purity of RNA was assessed using a Nanodrop spectrophotometer (ND-1000). One microgram of total RNA was reverse transcribed to cDNA using the Superscript III First-Strand cDNA Synthesis System for RT-PCR (Invitrogen; 18080–051). No-RT controls were processed in the same way as cDNA samples without the Superscript III enzyme.

### Quantitative real-time PCR (qRT-PCR)

Either 0.2 or 0.4% of the total cDNA volume was used in a SYBR green PCR assay (New England Biolabs; F-410 l). PCR was performed on a 7500 Fast Real-Time PCR System (Applied Biosystems). The PCR cycling conditions were described previously [19]. The specificity of amplification for each gene was confirmed by agarose gel electrophoresis, melting curve analysis and sequencing analysis. The cycle threshold (Ct) for each gene was determined by setting the Ct line at the center of the logarithmic phase of amplification for that particular gene. The Ct differences between mock-infected and virus-infected samples (analyzed in triplicate) were calculated using the delta delta Ct method [53]. Primer sets used for the amplification of IFN-β, ISG54 and β-actin were as previously described [19, 54].

### Immunoblotting

HEC-1B cell lysates were prepared by scraping cells into PBS followed by centrifugation at 200 × *g* for 5 min and resuspended in Tx lysis buffer (50 mM triethanolamine pH 7.4, 500 mM NaCl; 0.5 % Triton X-100; 1 mM dithiothreitol) containing 1X protease and phosphatase inhibitor cocktail (Calbiochem; 539134 and Sigma; P2850, respectively). Following a 20 min incubation on ice lysates were centrifuged at 16,000 *g* for 5 min and the pellet was discarded. Protein quantification was determined using the Bio-Rad protein assay kit. Equal quantities of protein were separated on SDS-8 % PAGE followed by transfer to a PVDF membrane (Millipore Corporation). Phosphorylated ATF-2 was detected using rabbit polyclonal and rabbit monoclonal antibodies, respectively (Cell Signaling; #9221). Rabbit polyclonal antibodies were used to detect IPS-1 (Axxora; ALX-210-929), MDA-5 (a kind gift from Dr. Paul Fisher) [55], IRF-3 and NF-κB/p65 (Santa Cruz Biotechnology;sc-9082 and sc-109, respectively) and β-actin (Abcam; ab8227). Antibody-antigen complexes were detected using an HRP conjugated secondary antibody and chemiluminescence.

### IRF-3 dimerization assay

Cell lysates were prepared by scraping cells into PBS followed by centrifugation at 200 × *g* for 5 min and resuspension in native PAGE lysis buffer (50 mM Tris-Cl pH 8.0, 150 mM NaCl and 1 % NP-40) containing 1X protease and phosphatase inhibitor cocktail. Non-denaturing 7.5 % acrylamide gels were pre-run at 40 mA constant current for 30 min in cathode buffer (25 mM Tris-Cl, 192 mM glycine and 0.2 % DOC, pH 8.4) and anode buffer (25 mM Tris-Cl,192 mM glycine, pH 8.4) at room temperature and equal quantities of proteins were separated at 25 mA constant current for 50 min at 4 °C [40]. Proteins were transferred to PVDF membrane at a constant current of 350 mA for one hour at 4 °C. IRF-3 monomers and dimers were detected using an anti-IRF-3 rabbit polyclonal antibody (Santa Cruz Biotechnology: sc-9082) followed by an HRP conjugated secondary antibody and chemiluminescence.

### Immunofluorescence

HEC-1B cells were seeded onto 12 mm diameter coverslips and 48 h later cells were either mock-infected or infected with poliovirus at an MOI of 50. Coverslips were removed at the indicated times, fixed in 3 % formaldehyde for 15 min at 25 °C, washed three times in PBS and permeabilized in methanol for 5 min at −20 °C. Coverslips were then washed 3 times in PBS, incubated in blocking solution (2 % BSA, 0.05 % Triton X-100 in PBS) for 30 min at 25 °C and incubated overnight at 4 °C in primary antibody against NF-κB/p65 (Santa Cruz Biotechnology: sc-109). Coverslips where then washed three times in blocking solution, incubated for 1 h at 25 °C in secondary antibody conjugated to AlexaFluor 555 (Life Technologies), washed two times in PBS, stained with Hoechst 33258 (0.2 μg/ml in PBS) and mounted on glass slides with Vectashield mounting medium (Vector laboratories; H-1000). Cells were viewed on a Nikon E1000M fluorescent microscope at 60X magnification and images were obtained using a Hamamatsu Orca 285 digital monochrome camera and Metamorph software (Universal Imaging).

### Poly(I:C) treatment

HEC-1B cells were seeded in 35 mm wells and were either mock-infected or infected with poliovirus at an MOI of 100. After 1.5 h cells were transfected with 100 μg/ml of poly(I:C) (Sigma; P0913) using 800 μg/ml of DEAE-dextran (Sigma; D9885) in serum free DMEM. Mock-treated cells were exposed to DEAE-dextran in the absence of poly(I:C). After an additional 1.5 or 2.5 h incubation total RNA and protein lysates were prepared. IFN-β mRNA levels and homodimerization of IRF-3 were quantified as described above.
